# Age and Maturation Stage Linked Consequences of Fibrinogen on Human Oligodendroglia

**DOI:** 10.1002/jnr.70120

**Published:** 2026-03-22

**Authors:** Gabriela J. Blaszczyk, Chao Weng, Abdulshakour Mohammadnia, Qiao‐Ling Cui, Arianna Giurleo, Adam M. R. Groh, Chloe Plouffe, Julien Sirois, Valerio E. C. Piscopo, Moein Yaqubi, Asad Taqvi, Erin Cassidy, Liam Callahan Martin, Jeffery A. Hall, Roy W. R. Dudley, Myriam Srour, Stephanie E. J. Zandee, Wendy Klement, Sandra Larouche, Alexandre Prat, Thomas M. Durcan, Jo Anne Stratton, Jack P. Antel, G. R. Wayne Moore

**Affiliations:** ^1^ Neuroimmunology Unit, Montreal Neurological Institute‐Hospital and Department of Neurology and Neurosurgery McGill University Montreal Quebec Canada; ^2^ Department of Neurology Renmin Hospital of Wuhan University Wuhan Hubei People’s Republic of China; ^3^ Early Drug Discovery Unit, Montreal Neurological Institute‐Hospital McGill University Montreal Quebec Canada; ^4^ Department of Neurology and Neurosurgery McGill University Health Centre (MUHC) and the Montreal Neurological Institute‐Hospital Montreal Quebec Canada; ^5^ Department of Pediatric Neurosurgery Montreal Children’s Hospital Montreal Quebec Canada; ^6^ Division of Pediatric Neurology Montreal Children’s Hospital Montreal Quebec Canada; ^7^ Centre de Recherche Hospitalier de L’université de Montréal Montreal Quebec Canada; ^8^ Department of Pathology and Laboratory Medicine University of British Columbia; International Collaboration on Repair Discoveries (ICORD) Vancouver British Columbia Canada; ^9^ Division of BioMedical Sciences Memorial University of Newfoundland St. John’s Newfoundland and Labrador Canada

**Keywords:** fibrinogen, human cell culture, myelination, oligodendrocytes, oligodendroglial precursors

## Abstract

Fibrinogen is a blood‐derived protein involved in coagulation and can make its way into the central nervous system (CNS) following breakdown of the blood–brain barrier. This molecule has been implicated in multiple sclerosis (MS), a disease marked by inflammation and demyelination in the CNS. However, the effect of this molecule has not been studied on human myelinating cells. This study examines how fibrinogen influences human oligodendrocyte (OL) lineage cells at various stages of development. Using induced pluripotent stem cell‐derived (iPSC) OL precursors and human primary OLs, we examined the effects of fibrinogen on cell differentiation, viability, and myelination‐related function. Here we show the differential effect of fibrinogen, based on OL‐lineage stage. While fibrinogen induced aberrant differentiation of early lineage OLs, by inhibiting their maturation and inducing an astrocytic phenotype, on mature OLs fibrinogen was found to promote myelination capacity, as shown by ensheathment assays as well as on the RNA level. These effects were associated with the activation of bone morphogenetic protein (BMP) signaling, both in early and mature OLs. We further found BMP signaling enrichment in OLs to be correlated with the inflammatory activity of an MS lesion and confirmed fibrinogen deposition on OLs in situ. Unlike previous rodent studies, these findings indicate that fibrinogen has a lineage‐dependent effect, where it may be inhibitory earlier in the lineage while promoting OL function in later stages. Understanding this dual role will provide insight into remyelination failure in MS and highlights the importance of timing and target in future therapeutic strategies.

## Summary

- Fibrinogen induces aberrant differentiation of human early lineage oligodendroglia, by inhibiting their maturation and inducing an astrocytic phenotype.
- Fibrinogen promotes myelination capacity in mature human oligodendroglia.
- These effects were associated with the activation of bone morphogenetic protein (BMP) signaling, both in early and mature oligodendroglia.
- There is BMP signaling enrichment in oligodendroglia evident in chronic active multiple sclerosis plaques.
- Fibrinogen deposition is evident on oligodendroglia in MS plaques and normal‐appearing white mater.

## Graphical abstract

This study shows that the blood‐derived molecule, Fibrinogen, has a differential effect on human Oligodendroglial (OL) cells. Fibrinogen, when added in vitro, leads to aberrant differentiation of OL‐precursors towards an astrocytic fate. On the other hand, when added into the culture of mature OLs, fibrinogen increases myelination capacity. Fibrinogen acts through the bone morphogenetic protein (BMP) pathway across the OL lineage, which is confirmed via fluorescence imaging and on the RNA level. This BMP pathway signature was found in situ on OLs derived from chronic active lesions, and fibrinogen was found to be in contact with OL‐lineage cells in both MS lesions and normal appearing white matter. This figure was created in BioRender. Durcan, T. (2026) https://BioRender.com/b0d3fb4 .

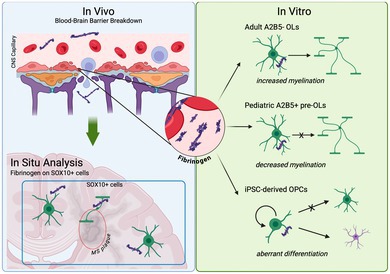

## 1 Introduction

Potential contributors to the injury and repair responses of oligodendroglia (OLs) are blood‐derived factors that may access the central nervous system (CNS) due to the breakdown of the blood–brain barrier (BBB) (Zierfuss et al. 2024). Fibrinogen is a circulating blood clotting factor composed of polypeptide chains which have polymerization sites for clot formation (Budzynski 1986), and various domains known to be involved in stimulation of multiple cell types (Mosesson 2005). Fibrinogen has been described in multiple sclerosis (MS) and detected in the borders of chronic active white matter plaques (Lee et al. 2018) and leakage into diffusely abnormal white matter (Vos et al. 2005) and normal‐appearing white matter (McQuaid et al. 2009). It has also been found in MS motor cortex, where its presence significantly correlated with reduced neuronal density (Yates et al. 2017), implicating its direct role in cellular damage. Moreover, plasma levels of fibrinogen are elevated in MS relapses (Çiçekli et al. 2022), and its role in MS pathogenesis has been linked to its function in the coagulation cascade and as a pro‐inflammatory agent (Alruwaili et al. 2023). The role of fibrinogen in experimental autoimmune demyelination in the CNS has been extensively studied (Ryu et al. 2015), and rodent studies have shown that fibrinogen can inhibit the differentiation of rodent oligodendrocyte precursors (OPCs) into myelinating oligodendrocytes, primarily mediated via bone morphogenetic protein (BMP) signaling (Petersen et al. 2017). While fibrinogen and related components of the coagulation process are thought to have an important role in neuroinflammation (Ryu et al. 2015; Bardehle et al. 2015) and the pathogenesis of MS (Alruwaili et al. 2023; Petersen et al. 2018; Davalos et al. 2019; Ziliotto et al. 2019; Ahmad and Frederiksen 2020), it is unknown if these findings are translatable to human oligodendroglia across their development.

The objectives of our study were to extend insights into the direct effects of fibrinogen on human OLs in relation to age and lineage stage. We employed primary human mature OLs (O4 + A2B5−) and late lineage cells (O4 + A2B5+) (Ruffini et al. 2004; Esmonde‐White et al. 2019) derived from surgically‐resected tissue samples together with early‐stage progenitor cells derived from human induced pluripotent stem cells (iPSCs), as such primary cells cannot currently be derived from available human CNS sources. For iPSC studies, we employed a previously characterized reporter line, where the degree of expression correlated with the differentiation stage of the cells (Piscopo et al. 2024). Primary cell studies were also considered in context of the donor age, as we previously reported that ensheathment capacity, as determined using cultures with synthetic nanofibers, and susceptibility to injury were donor age dependent (Luo et al. 2022). Using these methods, we found that adult‐derived mature OLs respond to fibrinogen by increasing their ensheathment capacity. While pediatric late‐stage progenitors, and iPSC‐derived early progenitors decrease their differentiation, in line with previous reports that studied various other toxic effects on rodent OLs (Ryu et al. 2015).

## 2 Materials and Methods

This study was ethically approved by the Institutional Review Board of the McGill University Faculty of Medicine and Health Sciences (A09‐M72‐22B; 22‐08‐053).

### 2.1 Antibodies

Antibodies used in this study are listed in Table S4.

### 2.2 Human Primary Cell Isolation and Culture

Studies were conducted on OLs derived from pediatric (2.5–18 years) and adult (26–65 years) tissue samples collected from patients undergoing surgery, regardless of biological sex. Further donor characteristics can be found in Table S1. The use of adult tissues was approved by the Montreal Neurological Institute Neurosciences Research Ethics Board and the use of pediatric tissues by the Montreal Children's Hospital Research Ethics Board. As previously described (Esmonde‐White et al. 2019; Luo et al. 2022), samples were dissociated by trypsin digestion and subjected to Percoll gradient (Sigma, Oakville, ON) separation. To enrich progenitor populations, MACS magnetic beads selection against the cell surface marker A2B5 (Miltenyi Biotec, Auburn, CA) was performed following cell isolation, and positive and negative fractions were cultured as described previously (Esmonde‐White et al. 2019; Luo et al. 2022).

Our previous detailed flow cytometry‐based analysis of the adult and fetal cell populations (Leong et al. 2014) indicated that of the OL population isolated from the adult brain 5%–10% of the cells were recognized by the A2B5 antibody (referred to as A2B5+ cells) and are considered late progenitor cells. 85%–90% of isolated OL cells express detectable levels of O4. The two cell fractions (A2B5+ and A2B5−) express comparable levels of mature myelin genes (*MBP* and *PLP*) based on bulk RNA sequencing, whereas progenitor genes (*PDGFRA* and *PTPRZ1*) are more highly expressed in the A2B5+ cells (figure 3 in Luo et al. (2022)).

#### 2.2.1 Viability Assays in Dissociated Cell Cultures

The cells were cultured in DMEM/F12+ N1 medium +0.1% BSA + Pen/Strep + B27 medium (Life Technologies, Grand Island, NY) in 96 well plates (Fisher Scientific) and treated with plasminogen‐depleted fibrinogen (Millipore Sigma, Burlington MA, 341578, 80ug/ml to 4 mg/mL) for 6 days, with addition of fibrinogen alongside media changes every other day. Cells were stained live for O4 (1:200, R&D systems, Oakville, ON) and the cell viability was determined by live staining with propidium iodide (PI, 1:200, Thermo Fisher Scientific, Mississauga, ON).

### 2.3 Nanofiber Ensheathment Cultures

Aliquots of A2B5+ and A2B5− cells were plated in multi‐well aligned poly‐L lactide nanofiber plates (The Electrospinning Company Ltd., Didcot, Oxfordshire, UK) (10,000 cells per well) and treated with plasminogen‐depleted fibrinogen (2.5 mg/mL) in DMEM/F12+ N1 medium supplemented with B27 (Life Technologies, Grand Island, NY) and T3 (Sigma‐Aldrich, Oakville, ON) for 2 weeks. Fibrinogen was supplemented alongside media changes every other day. Cell cultures were immunolabeled with O4 antibody diluted 1:200 (R&D Systems, Oakville, ON) for 1 h, followed by the corresponding secondary antibody (1:500) conjugated with Alexa Fluor 647 (Thermo Fisher Scientific, Mississauga, ON) for 2 h. To determine the percentage of O4+ cells ensheathing nanofibers in a culture, a blinded rater counted individual O4+ cells whose nanofibre‐ensheathing processes could be identified as being connected to their cell bodies identified by Hoechst 33,342 (Thermo Fisher Scientific, Mississauga, ON) staining of nuclei. Ensheathed processes were recognized by their straight alignment with underlying nanofibers and increased thickness of the segments (Figure 2D,E). Cells were assigned as ensheathing (one or more segments) or not and counted by the Plugin Cell Counter ImageJ program.

### 2.4 Human iPSC Culture and OPC Differentiation

iPSC‐derived OL lineage cells were differentiated according to a recently published protocol (Piscopo et al. 2024). The use of iPSCs and stem cells in this research was approved by the McGill University Health Centre Research Ethics Board (DURCAN_IPSC/2019–5374). iPSCs were used from healthy control lines as well as the SOX10‐mOrange reporter line (Piscopo et al. 2024), which were generated at the Montreal Neurological Institute's (MNI) Early Drug Discovery Unit (EDDU) (Table S2). According to a previously published protocol (Piscopo et al. 2024), early OL‐lineage progenitors were generated and plated on poly‐L‐ornithine (Sigma‐Aldrich, Oakville ON)/laminin (Invitrogen, Waltham MA) coated culture vessels. Post day 75 in vitro, cells are used for up to a maximum of four passages and differentiated to reach a majority of OL‐lineage cells at a total of 21 days. As regards the composition of iPSC derived cells, at post day 75 in vitro (OPC phase) approximately 50% of the cells express the early marker PDGFRα and approximately 36% the ganglioside A2B5. Between 20% and 40% of the total cells express O4 (Piscopo et al. 2024).

Cells were treated with plasminogen‐depleted fibrinogen (Millipore Sigma, Burlington MA, 341578, 2.5 mg/mL) for 4 days prior to fixation (days 18–21 of differentiation, in differentiation medium). The cell viability (PI) and differentiation (O4) were determined as described above. Proliferation was determined by staining with Ki67 (Invitrogen, Waltham MA), post‐fixation and permeabilization diluted 1:200, overnight. Secondary antibody (Alexa Fluor 488 or 647; Invitrogen, Waltham MA) was applied the following day, diluted to 1:400.

### 2.5 Immunofluorescence Analysis

Plates were imaged with a 10× objective using a Zeiss Axio Observer fluorescence microscope (Carl Zeiss Canada, Toronto) or the ImageXpress (Molecular Devices, San Jose, CA) high‐content imaging platform following staining for O4, PI or Ki67. Cells were counted by a blinded individual using the ImageJ software or automatically using the ImageXpress software.

### 2.6 Signaling Assessment

#### 2.6.1 Staining

Primary human OLs and iPSC‐derived OPCs (following 21 days of differentiation) were treated with molecules for 2 h to induce signaling. The following concentrations were applied: plasminogen‐depleted fibrinogen (Millipore Sigma, Burlington MA, 341578, 2.5 mg/mL), recombinant human BMP4 (Peprotech, Rocky Hill NJ, 50 ng/mL), BMP4‐inhibitor DMH1 (Selleck Chemicals, Houston TX, 2 uM). Post‐fixation, cells were permeabilized with 100% cold methanol and stained with anti‐phosphorylated (p)SMAD1/5/9 (Cell Signaling Technology, Danvers MA) overnight at a 1:50 dilution, and secondary antibodies (Alexa Fluor 488, 555 or 647; Invitrogen, Waltham MA) diluted to 1:400 were added for 2 h the following day.

#### 2.6.2 To Measure Mean Fluorescence Intensity (MFI)

Images were acquired on a Zeiss Axio Observer fluorescence microscope (Carl Zeiss Canada, Toronto, ON) at 20X objective or the Zeiss LSM 900 confocal microscope at 20× objective. For pSMAD1/5/9 nuclear intensity, nuclei of O4+ cells were selected by a blinded individual on the ImageJ software and the mean gray value of the pSMAD1/5/9 channel was recorded.

#### 2.6.3 To Record pSMAD Localization

Following image acquisition at the 20X objective, localization of pSMAD1/5/9 on O4+ cells was assessed by a blinded individual based on four criteria: no signal, cytoplasm only, nucleus and cytoplasm, nuclear only. 20–25 cells were counted for each replicate.

### 2.7 FACS Sorting and Flow Cytometry

Using the iPSC‐reporter line SOX10mO (Piscopo et al. 2024), cells were collected from the culture vessel using TrypLE (Thermo‐Fisher, Mississauga, ON) added for 30 min at 37°C. The cells were passed through a 40 μM mesh to ensure a single cell suspension. Following resuspension in PBS (Wisent, Saint‐Jean‐Baptiste, QC), the cells were counted using the Attune flow cytometer (Thermo‐Fisher Scientific, Mississauga, ON). Sorting was based on reporter intensity using the previously described protocol (Piscopo et al. 2024), with the Aria fusion cell sorter (BD Biosciences, Franklin Lakes NJ). For phenotyping, cells were stained as previously described (Piscopo et al. 2024), and acquired on the Attune flow cytometer (Thermo‐Fisher Scientific, Mississauga, ON). Data was analyzed using the FlowJo software (Ashland, OR).

### 2.8 Bulk RNAseq Preparation and Analysis

Three subsequent passages of FACS‐sorted SOX10‐mOrange cells were collected as replicates, and primary cells from two different donors (53 and 58 years) were collected as replicates following treatment with plasminogen‐depleted fibrinogen (Millipore Sigma, Burlington MA, 2.5 mg/mL) for four and 2 days, respectively. Treated cells were collected, and RNA was extracted using the Qiagen (Hilden, Germany) RNA‐mini kit, or the Norgen (Thorold, ON) single cell RNA purification kit.

#### 2.8.1 Library Preparation, Sequencing, Quality Check, Alignment, Quantification of Raw Read Counts

Library preparation, sequencing, quality check, alignment, quantification of raw read counts, and normalization of read counts were performed using the same methods as described in Mohammadnia et al. (2024).

#### 2.8.2 Analysis of RNA‐Seq Data From Human Primary Oligodendrocytes

Samples were analyzed in a pairwise manner as described in Mohammadnia et al. (2024).

#### 2.8.3 Analysis of RNA‐Seq Data From iPSC‐Differentiated Cells

As these samples did not exhibit significant heterogeneity and the lack of pair information for samples, we used DESeq2 for differential gene expression analysis following the methodology outlined in Pernin et al. (2024) (Love et al. 2014; Pernin et al. 2024). Significantly differentially expressed genes were identified using a threshold of log2 fold change > 1 and a *p*‐value cutoff of < 0.05.

Heatmaps were generated following hierarchical clustering using the “Hierarchical Clustering Image” function in GenePattern (v3.9.11). Gene clustering was performed using Pearson correlation, and normalized values were transformed using a log transformation. Finally, row normalization was applied for the final visualization (Reich et al. 2006). Single‐sample Gene Set Enrichment Analysis (ssGSEA) was performed using the same method described in Mohammadnia et al. (2024). However, the signatures used in this analysis were BMP4 target genes extracted from the weighted matrix of ligand‐target interactions in NichNet (Browaeys et al. 2020). We selected target genes based on their weighted values, specifically those with a weight > 0.1 (*n* = 107 genes). Volcano plots were generated in Python using pandas/numpy for data handling and matplotlib for visualization, plotting log2 fold change versus −log10 (*p*‐value). Genes were divided as upregulated (log2FC > 0.58 and *p* < 0.05), downregulated (log2FC < −0.58 and *p* < 0.05), or not significant, with dashed threshold lines drawn at these cutoffs.

#### 2.8.4 Single‐Nuclear RNA‐Seq Analysis

All single‐nuclear RNAseq analyses were performed using the same pipeline employed in our previous publication (Mohammadnia et al. 2025) on the same Absinta's dataset (Absinta et al. 2021).

### 2.9 Human MS Brain Tissue

Tissues from the Centre Hospitalier de l'Université de Montréal (CHUM) were obtained by a Material Transfer Agreement between CHUM and McGill University. Tissue samples were collected from three MS patients (Table S3) with full ethical approval (BH07.001, Nagano 20.332‐YP) and informed consent as approved by the local ethics committee. Human brain tissue was obtained from patients diagnosed with MS according to the revised 2010 McDonald's criteria (Polman et al. 2011). Donor characteristics are supplied in Table S3. Autopsy samples were frozen and lesions classified by the classic system into active (or acute), chronic active, and chronic inactive plaques (Moore and Stadelmann‐Nessler 2015) using Luxol Fast Blue‐Hematoxylin and Eosin (LFB‐HE) staining as previously published (Dhaeze et al. 2019; Broux et al. 2020).

### 2.10 Immunohistochemistry

Frozen human brain tissue sections, previously briefly fixed in acetone, were further fixed in 100% acetone for 10 min, followed by 70% ethanol for 5 min. Subsequently, sections were washed with PBS and 0.05% PBST (phosphate‐buffered saline + Tween20) for 3 min. Fc‐receptors in sections were blocked with PBS containing 10% donkey serum. Primary antibodies, goat anti‐SOX10 (R&D systems, Oakville ON, AF2864) and rabbit anti‐fibrinogen (Agilent, Santa Clara CA, A0080) were diluted 1:500 (0.2 mg/mL) and 1:400 (1 mg/mL) respectively in PBS containing 3% donkey serum and incubated overnight at 4°C. Isotype controls consisted of the IgG of the same species and same concentration as each primary antibody. Following incubation, sections were washed with 0.05% PBST 3 times for 5 min each. Fluorophore‐conjugated secondary antibodies (Invitrogen, Waltham MA) were prepared at a 1:200 dilution in PBS, donkey anti‐goat IgG Alexa Fluor 488 to visualize SOX10 and donkey anti‐rabbit IgG Alexa Fluor 647 to visualize fibrinogen and slides were incubated in the dark for 60 min at room temperature. Sections were washed with 0.05% PBST 3 times for 3 min and incubated with Hoechst (Invitrogen, Waltham MA; 1:5000) for 10 min at room temperature. Once washed with 0.05% PBST 3 times for 3 min, to block lipofuscin autofluorescence sections were incubated with 0.3% TrueBlack (Biotium, Fremont, CA) diluted in 70% ethanol for 3 min. Slides were washed with PBS and mounted with PermaFluor mounting medium (Thermo‐Fisher, Missisauga, ON), coverslipped and stored in the dark at 4°C.

### 2.11 Statistical Analysis

Statistical analyses were performed using Excel or GraphPad Prism software. Cell culture studies were performed with at least three individual replicates per experiment. For primary cultures, these are three individual donors, with each donor serving as the paired untreated control. For iPSC‐studies, three cell lines were used which contributed to values on graphs, and subsequent passages were used as experimental replicates. Bars on graphs indicate means ±SD. Lines on graphs represent matching and are further specified in the figure legends. Student's *t*‐test was used for comparisons between two groups, and multiple comparisons were corrected for using Benjamini‐Hochberg following 2‐way ANOVA. *p*‐values < 0.05 were considered statistically significant. Normality of values was ascertained using the Shapiro–Wilk test, which was non‐significant unless specified.

## 3 Results

### 3.1 Fibrinogen Inhibits the Differentiation of Human OPCs

**FIGURE 1:**
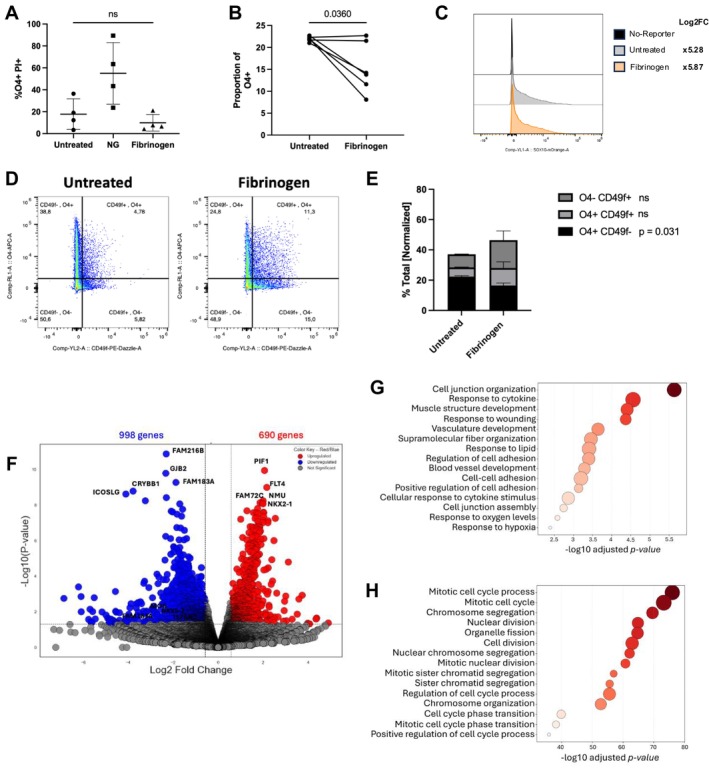
Fibrinogen effect on human iPSC‐derived OPCs. (A) Proportion of viable OPCs (O4+) following immunofluorescence staining for propidium iodide (PI). NG: No glucose. Two‐tailed paired *t*‐test to untreated control of each replicate: Fibrinogen versus untreated, *p* = 0.1142. Bars indicate SD. (B) Proportion of OPCs (O4+) as quantified following immunofluorescence microscopy, showing a decrease in OPCs following fibrinogen treatment for 4 days. Two‐tailed paired *t*‐test, *p* = 0.0360. Lines indicate matched values. (C) Representative histogram of the fluorescence intensity of the SOX10mO reporter. Log2 fold‐change of mean fluorescence intensity calculated relative to non‐reporter control, values shown are an average of *n* = 5 passages. *p* = 0.783 following paired *t*‐test. (D) Flow cytometry gating technique. (E) Proportions of live cells co‐expressing OPC/astrocyte markers as measured by flow cytometry, as gated in figure D. Values normalized to the average of the untreated control, *n* = 3 passages. Two‐tailed paired *t*‐tests were applied to assess significance levels in all groups. (F) Differentially expressed genes following bulk RNAseq analysis of sorted SOX10mO‐med cells. (G, H) GO term analysis of (G) downregulated and (H) upregulated genes. Significant GO terms were determined with a 1.5 log2FC cut off, and a *p* < 0.05.

To corroborate previous findings in rodents (Petersen et al. 2017), we aimed to test the effects of fibrinogen on human oligodendrocyte progenitors. Since, as stated, we cannot isolate early OPCs from our surgical samples, we generated early OL‐lineage cells from iPSCs. We also utilized a previously described human iPSC reporter line (SOX10mO) (Piscopo et al. 2024) where fluorescence intensity of the reporter correlates to the overall maturity of the cells. Fibrinogen treatment of early lineage cells had no significant effect on OPC viability (Figure 1A), although treatment did significantly reduce the proportion of O4+ OPCs (Figure 1B, *p* = 0.0360, paired *t*‐test) as measured by immunofluorescence (representative images in Figure S1). To assess the effect of fibrinogen on overall differentiation into the OL lineage, we assessed level of our SOX10mO reporter. We did not observe a significant change in the Mean Fluorescence Intensity (MFI) of SOX10mO (Figure 1C, *p* = 0.783, paired *t*‐test). However, after gating on our previously determined SOX10mO subgroups (Piscopo et al. 2024) (Figure S2), addition of fibrinogen resulted in an increase proportion of SOX10‐med cells, although non‐significant post False discovery rate (FDR) correction. We used the astrocyte‐specific marker CD49f (Barbar et al. 2020) to follow up on the observation by Petersen et al. (Petersen et al. 2017) that fibrinogen diverts the differentiation of primary post‐natal rodent derived OPCs towards the astrocyte lineage. We gated live cells on the expression of the OPC marker O4, against the astrocyte marker CD49f (Figure 1D). We observed that the overall decrease in O4+ cells (*p* = 0.031, paired *t*‐test) was associated with an increasing trend in proportion of O4‐CD49f + cells and O4 + CD49f + cells following fibrinogen exposure (Figure 1D,E). The latter hybrid OPCs retained the expression of our reporter (Figure S3).

To explore the molecular response of our SOX10mO cells, they were FACS‐selected as previously described (Piscopo et al. 2024). Top five upregulated protein‐coding genes (DEG's) include: *PIF1*, *FLT4*, *FAM72C*, *NMU*, and *NKX2‐1*. (log2FC > 2, Figure 1F and File S1). Top 5 downregulated protein‐coding genes by log2FC include; *FAM216B, GJB2, CRYBB1*, *ICOSLG*, and *FAM183A* (log2FC > 2, Figure 1F and File S1). Bulk RNAseq analysis indicates an overall decrease in cell–cell adhesion, and cell junction organization occurring in these early OL‐lineage cells (Figure 1G and File S2), which are important biological processes in myelination and OPC differentiation (Jagielska et al. 2012). OPC‐identity genes and late OL‐lineage genes such as *MOG* (*p* = 0.0048, File S1 and Figure S4) were downregulated, supporting our findings of decreased O4+ cell proportions in culture. Alongside this, significant upregulation of cell‐cycle pathways (Figure 1F–H), confirmed by Ki67 staining (Figure S5) was also observed. These findings coincide with findings by Petersen et al. (Petersen et al. 2017) in primary rodent OPCs and serve as confirmation for fibrinogen acting on OPC differentiation capacity in a human context.

**FIGURE 2:**
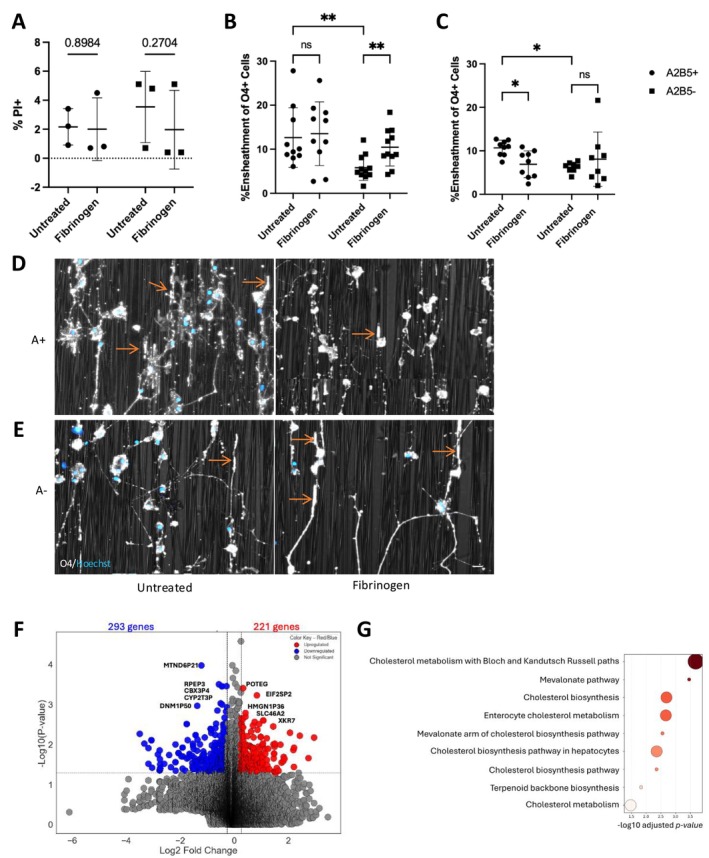
Fibrinogen has an age and maturation‐dependent effect on human OLs. (A) Cell viability of human A2B5+ pre‐OLs (A+) and A2B5− mature OLs (A−) from both adult and pediatric samples following fibrinogen treatment and quantified following immunofluorescence microscopy for propidium iodide (PI). Bars indicate SD. 2‐way ANOVA was insignificant, and post hoc Fisher's LSD test proved non‐significant results. Post hoc test was uncorrected as each comparison stands alone. (B, C) Capacity of (B) adult (26–65 years; 17 samples) and (C) pediatric (2.5–18 years; 14 samples) pre‐OLs (A2B5+ (A+)) and mature OLs (A2B5− (A−)) to ensheath nanofibers. *p < 0.05, **p < 0.01, ***p < 0.001. Bars indicate SD. 2‐way ANOVA was performed for each plot, resulting in *p*‐values of *p* = 0.004 and *p* = 0.006, respectively. Post hoc analysis and correction was performed with Fisher's LSD test. Adult (B) cells are as follows: Untreated samples have a significant difference from each other (0.0071), and fibrinogen treated A2B5− cells have a significant difference as well (0.0078), while A2B5+ cells do not (0.5938). Pediatric cells (C) are as follows: Untreated samples have a significant difference from each other (0.0158), and fibrinogen treated A2B5+ cells have a significant difference as well (0.0486), while A2B5− cells do not (0.3346). (D, E) Representative images of human OLs on nanofibers, cells derived from a 10 year old male. O4 stain in white, nuclei in blue. (D) top panels represent A2B5+ cells, (E) bottom panels represent A2B5− cells. Arrows point to representative examples of ensheathing segments. Representative images were cropped, and brightness was increased to best show areas of interest. (F) Volcano plot to visualize top upregulated and downregulated genes. (G) GO term analysis on upregulated genes following bulk RNA sequencing on fibrinogen‐treated adult OL samples, GO terms were determined to be significant with a 1.5 log2FC cut off, and a *p* < 0.05.

### 3.2 Human Primary OLs Have an Age and Maturation‐Dependent Functional Response to Fibrinogen

To further explore the effect of fibrinogen across the OL lineage, dissociated human primary OL cultures comprised of 95% mature OLs (Luo et al. 2022), were used. Following treatment, we observed no significant effect on cell viability (following 2‐way ANOVA and Fisher's LSD) (Figure 2A). To determine a functional effect on OLs, we assessed their capacity to ensheath poly‐L‐lactide nanofibers. We separated pre‐myelinating OLs (A2B5+) from mature OLs (A2B5−) to determine the relation of lineage maturity. In the presence of fibrinogen, we observed an increased capacity in adult‐derived mature OLs (A2B5−) (*p* = 0.0071, Fisher's LSD) but no effect on adult A2B5+ cells (*p* = 0.5938, Fisher's LSD) (Figure 2B).

Previous studies in our lab suggest that pediatric‐derived primary A2B5+ cells most closely resemble a precursor (Luo et al. 2022). Therefore, to extend our studies across the entirety of the OL lineage, we next sought to determine the effect of fibrinogen on pediatric donor cells. Following exposure, we observed a significant decrease in ensheathing A2B5+ cells (*p* = 0.0486, Fisher's LSD) but not mature OLs (A2B5−; *p* = 0.3346, Fisher's LSD) in comparison to the age‐matched untreated controls (Figure 2C). Representative images are shown in Figure 2D,E. To confirm our in vitro findings, we performed Bulk RNAseq analysis of adult OLs, where analysis was performed in a paired manner where each donor served as their own control due to genetic heterogeneity of human samples. Top upregulated protein‐coding genes include: *POTEG*, *EIF2SP2*, *HMGN1P36*, *SLC46A2*, and *XKR7* (log2FC > 1.5, Figure 2F and File S3). The transcriptome of adult derived mature OLs treated with fibrinogen shows significant upregulation of lipid metabolism pathways, including cholesterol synthesis (Figure 2G; Figure S6; File S4), consistent with this beneficial effect of fibrinogen on ensheathment by adult OLs.

### 3.3 Fibrinogen Signaling in Human OL Lineage Cells via the BMP Pathway

**FIGURE 3:**
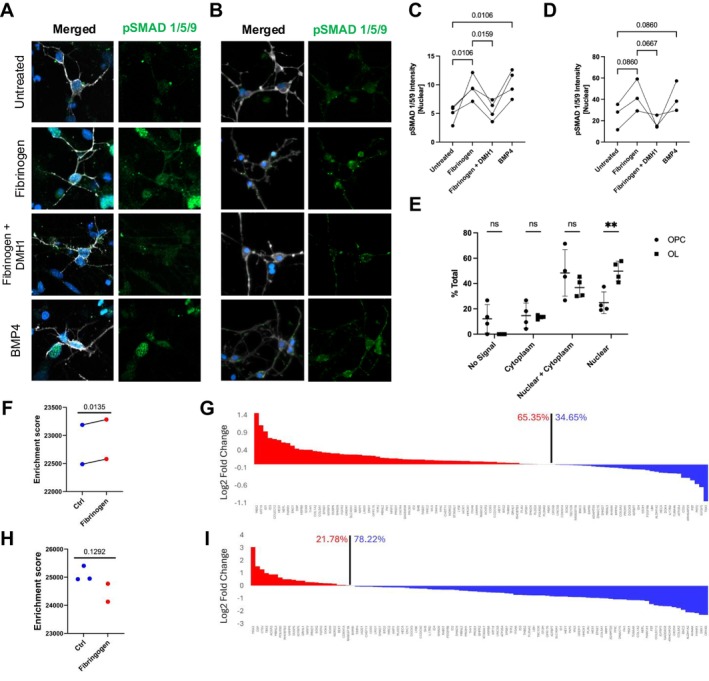
Fibrinogen activates BMP signaling across the OL lineage. (A, B) Representative images of (A) human iPSC derived OPCs (O4+) and (B) human primary OLs (O4+) following exposure to treatments for 2 h, by Zeiss confocal microscope. pSMAD 1/5/9 (green), O4 (white), nuclei (blue). Representative images were cropped and brightness was increased to best show areas of interest. (C, D) Quantification of nuclear fluorescence intensity of pSMAD 1/5/9 in (C) iPSC OPCs and (D) human primary OLs, where fibrinogen and BMP4 significantly increase pSMAD1/5/9 nuclear signal intensity. 1‐way ANOVA was performed between columns, and resulted in *p*‐values of (C) 0.0037 and (D) 0.0534. Post hoc testing was normalized using the Benjamini and Hochberg FDR method, and *p*‐values are shown above the bars on the graphs. (E) Proportion of pSMAD signal localization following treatment with fibrinogen, each point an average count of 25 cells in 1 biological replicate. *n* = 3 donors for primary cells, *n* = 4 passages for iPSC. 2‐way ANOVA resulted in a *p*‐value of 0.0025, and post hoc correction was performed with Benjamini and Hochberg FDR resulting in a *p*‐value of: No signal 0.0899, Cytoplasm 0.8650, Nuclear + Cytoplasm 0.1046, Nuclear 0.0013. Bars indicate SD. (F, H) Single‐sample Gene Set Enrichment Analysis (ssGSEA) for BMP4 target genes in: (F) human primary OLs treated with fibrinogen at 2.5 mg/mL, 2 days and (H) iPSC‐differentiated SOX10‐positive medium cells treated with fibrinogen at 2.5 mg/mL for 4 days. Normalized read counts were used for both analyses. Enrichment scores are visualized in the plots. One iPSC‐OPC fibrinogen‐treated sample was removed during quality control. (G, I) Bar plot showing log2‐transformed fold changes (ratios) of BMP4 target gene expression in (G) human primary OLs and (I) iPSC‐derived OPCs. Red bars indicate genes with higher expression levels in fibrinogen‐treated cells, and blue bars indicate genes with higher expression levels in the control group.

Fibrinogen has been previously suggested to act via the BMP pathway in rodent OL lineage cells, and inhibiting this pathway abrogates its effect (Petersen et al. 2017). BMP ligands have been previously shown to induce astrogenesis in OPCs and their precursors (O2A cells) (Mabie et al. 1997; Grinspan et al. 2000), so BMP pathway activation in earlier OL‐lineage could explain the effect observed (Figure 1). To determine if the BMP pathway was activated in human cells throughout the OL lineage in response to fibrinogen, we measured the activation of a downstream signaling factor, SMAD1/5/9 (Heldin et al. 1997). Nuclear localization of phosphorylated SMAD (pSMAD), as opposed to cytoplasmic, indicates activation, and we used this as a readout for our study. To confirm pathway activation earlier in the OL lineage, human iPSC‐derived OPCs were exposed to fibrinogen for 2 h, with representative images shown in Figure 3A, and mean fluorescence intensity (MFI) quantified in Figure 3C (1‐way ANOVA *p* = 0.0037). We observed a significant increase in nuclear localization of pSMAD1/5/9 in response to fibrinogen (Figure 3C) in comparison to untreated cells (*p* = 0.0053, Benjamini‐Hochberg). In a similar manner, we evaluated signaling in the primary OLs, with representative images in Figure 3B, quantified in Figure 3D (1‐way ANOVA *p* = 0.0534). We observed a similar trend in the OLs as in the iPSC, albeit insignificant following correction for multiple comparisons (*p* = 0.0690, Benjamini‐Hochberg). For both primary adult and iPSC‐derived O4+ cells, BMP4 treatment induced similar changes and fibrinogen‐induced responses were significantly reduced by the BMP‐pathway inhibitor, DHM1 (Figure 3). Comparing the early OPCs and the mature OLs (2‐way ANOVA *p* = 0.0025; Figure 3E), nuclear pSMAD localization was more frequent in the latter (*p* = 0.0013, Benjamini‐Hochberg), indicating that the BMP pathway is more activated in these cells than iPSC‐derived cells after exposure to fibrinogen.

Confirming these findings transcriptomically, bulk RNA seq analyses of fibrinogen‐treated mature OLs show enrichment of BMP‐regulated genes (Figure 3F,G) following ssGSEA. Individual BMP‐regulated genes also showed an increasing trend in the fibrinogen‐treated samples (Figure S7). Regarding the early OL‐lineage cells, we detected a non‐significant but decreasing trend in BMP pathway enrichment (Figure 3H,I). We did not observe significant changes in BMP‐regulated genes including those of the astrocyte lineage (data not shown). The early OL‐lineage cells showed an overall gene suppression except for an increase in cell proliferation related pathways (Figure 1F–H), which could explain the lack of BMP‐regulated genes observed. These data show that although fibrinogen activates the BMP pathway in vitro both in early and mature OL‐lineage cells, the downstream genes which are activated remain different.

### 3.4 OLs in MS Have Enriched BMP Signaling and Co‐Localize With Fibrinogen

**FIGURE 4:**
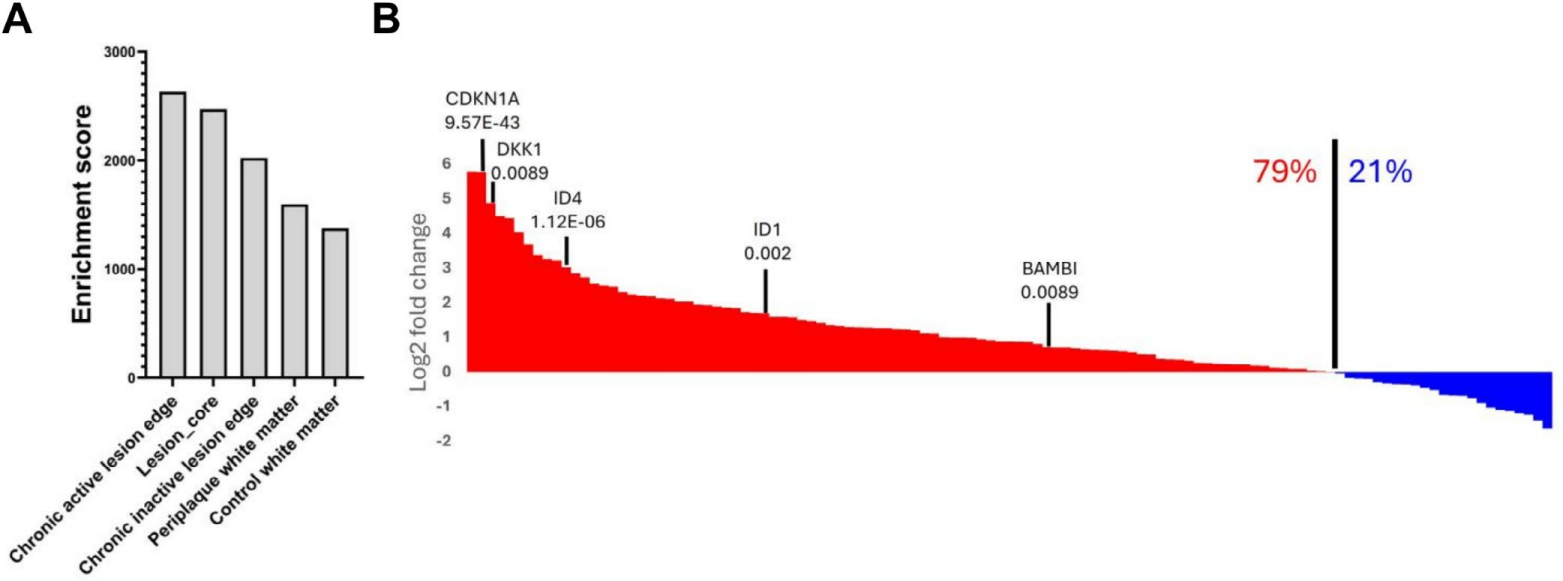
BMP signaling enrichment is found to correlate with degree of inflammation in the MS brain. (A) Single‐sample gene set enrichment analysis (ssGSEA) was performed on mature oligodendrocytes derived from different lesion types (Absinta et al. 2021). To run ssGSEA, BMP4 target genes (119 genes) were used as the gene set, and average expression levels for each lesion type were used as the reference. (B) Markers identified between oligodendrocytes from chronic active lesions and control white matter (from control patients). Five BMP signaling target genes and their corresponding *p*‐values were visualized. Genes were ordered based on fold changes for the visualization.

**FIGURE 5:**
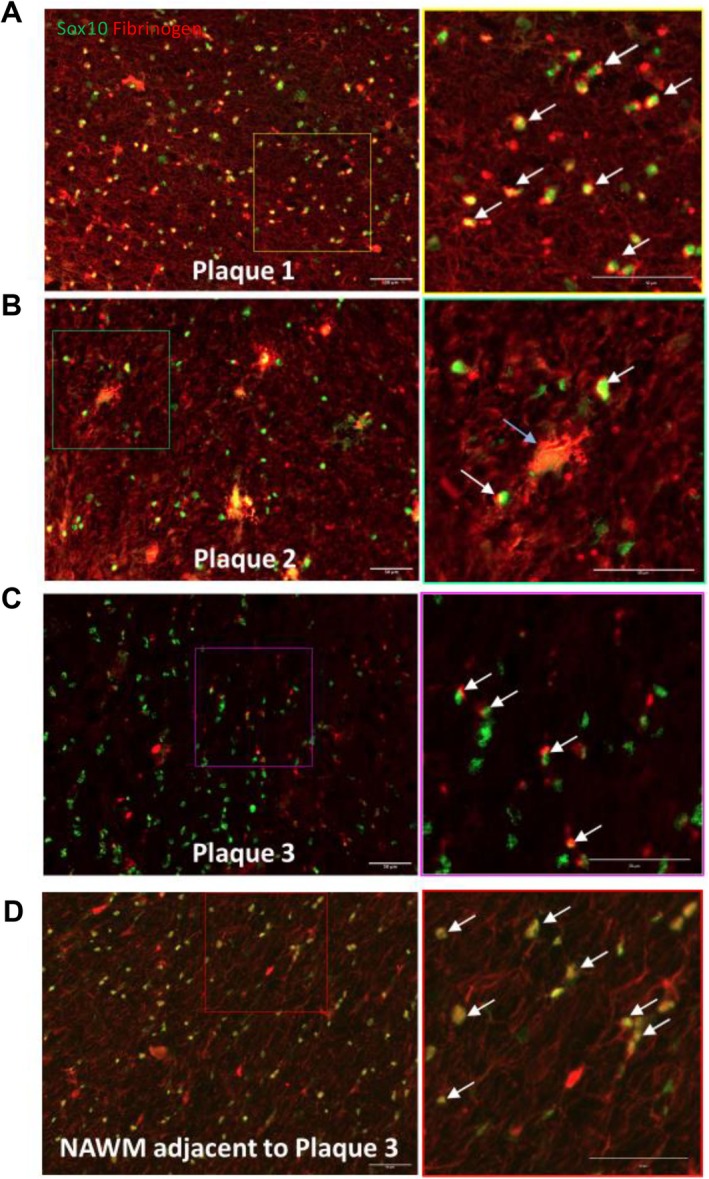
Colocalization of fibrinogen and OL lineage cells in the MS brain. Representative images of MS plaque and MS NAWM. Sox10 (oligodendroglial nuclear marker) in green; fibrinogen in red. For each region, a higher magnification of the area within the box is shown in the panel immediately to the right. (A) Plaque 1, an MS lesion that in sections stained for myelin showed active demyelination at its edge, consistent with a chronic active plaque; (B) Plaque 2, an MS lesion that in sections stained for myelin showed no active ongoing demyelination, consistent with a chronic inactive plaque. (C) Plaque 3, an MS lesion that in sections stained for myelin showed active demyelination throughout its extent, consistent with an active plaque. (D) The representative NAWM illustrated is adjacent to Plaque 3. Examples of SOX10‐positive oligodendroglia that are positive for fibrinogen in their cytoplasm and/or nuclei are indicated with white arrows; the cytoplasmic SOX‐10 staining suggests translocation from the nucleus in some oligodendroglia with fibrinogen. The blue arrow indicates a prominent fibrinogen‐positive cell, possibly an astrocyte or macrophage that has engulfed SOX10‐ and fibrinogen‐positive debris. Scale bars = 50 μm.

To ascertain in vivo relevance of our findings, we re‐analyzed the transcriptomic profiles from the Absinta's dataset (Absinta et al. 2021). In this dataset, information regarding the localization of the sequenced OLs was used in subsequent analyses. We found BMP signaling signatures in OLs, most predominantly found in chronic active lesions (Figure 4A), with the lowest enrichment score in OLs from control donor tissue. Following ssGSEA of OLs from chronic active lesions in comparison to control tissue, we found a 79% upregulation of the BMP signature, with many significant BMP target genes (Figure 4B). To determine if fibrinogen co‐localizes with OLs in the brain, tissue sections of the MS brain were co‐immunostained with SOX10 as a marker of OL lineage cells (Kuhlbrodt et al. 1998) and fibrinogen (Figure 5). SOX10 was used as it is expressed by all cells committed to the OL lineage, and therefore we could evaluate colocalization of fibrinogen with the entirety of the lineage. We stained a chronic active (Figure 5A, plaque 1), chronic inactive (Figure 5B, plaque 2), an active lesion (Figure 5C, plaque 3), and normal appearing white matter (NAWM, Figure 5D) to determine fibrinogen distribution across different stages of MS lesion formation and repair. We observed fibrinogen on SOX10‐positive oligodendroglia in MS lesions and adjacent white matter, with great variability between lesions (Figure 5).

## 4 Discussion

Fibrinogen, a blood coagulation protein, is hypothesized to be deposited in many CNS diseases with BBB disruption, including MS, stroke, traumatic brain injury, primary and secondary CNS tumors, infarcts, hemorrhages, and infectious processes. In neurodegenerative conditions, such as Alzheimer's disease, Parkinson's disease, Huntington's disease and ALS, fibrinogen has been found to be deposited in the CNS and has been thought to be important in their pathogenesis (Wen and Zhang 2023). Its involvement throughout the MS disease course is highlighted by studies showing disruption of the BBB and fibrin deposition early in MS lesions and preceding demyelination (Marik et al. 2007), while in progressive MS with severe cortical fibrinogen deposition, there is reduced neuronal density (Yates et al. 2017).

Fibrinogen has been noted to have a functional effect on immune cells, and resident CNS glia such as astrocytes and microglia (Petersen et al. 2018; Adams et al. 2007; Schachtrup et al. 2010). This molecule has been tested on rodent OPCs and OLs (Petersen et al. 2017), but, to our knowledge, no studies have documented the effect of fibrinogen on OL‐lineage cells in human samples. Here we demonstrate the in vitro effect of fibrinogen on human OL‐lineage cells is dependent on differentiation stage and provide evidence of co‐localization of fibrinogen on OL‐lineage cells in MS brain tissue (Figure 5).

As our primary cell isolation technique is not conducive to isolation of early lineage cells (PDGFR⍺ + A2B5+ O4+), we derived OPCs from human iPSCs and employed a previously characterized iPSC reporter line (SOX10mO) to aid in determining the overall stage of maturation in our culture (Piscopo et al. 2024). In line with previous rodent studies (Petersen et al. 2017), we found reduced differentiation capacity of OPCs (Figure 1B–E and Figure S4). This data is further compatible with the inhibition of ensheathment by pediatric A2B5+ cells, the most progenitor‐like cells within our primary cell isolates (Figure 2C). Concurrently, we observed an increase in proliferation of our cells, as seen in the increase in proportion of SOX10‐med cells (Figure S2), increased Ki67 (Figure S5), and upregulated GOBP terms (Figure 1H). The decrease in proportion of O4+ cells was associated with the increase in cells co‐expressing the astrocyte surface marker, CD49f (Figure 1D,E). This deviation towards the astrocyte lineage parallels with the findings of Petersen et al. (Petersen et al. 2017) in their rodent study, and was confirmed by expression of our reporter (Figure S3). However, an astrocytic gene signature was not evident in our bulk RNAseq data. This may be due to this being a small sub‐population of cells less prominent within the larger SOX10‐med sample, where overall cell cycling genes were more predominantly upregulated. We hypothesize that longer exposure to fibrinogen may be necessary to exhibit this effect on the wider population and may be more representative of the chronic exposure in vivo. Further exploration would be required to answer this question.

The mature OL plays important roles in myelin maintenance and can even contribute to re‐myelination in the post‐natal brain (Yeung et al. 2014). To assess the functional effects of fibrinogen on OLs in relation to their stage in the cell lineage, we obtained mature human OLs from surgically resected materials as described previously (Ruffini et al. 2004; Esmonde‐White et al. 2019). These samples were characterized further into late progenitors (A2B5+) and mature OLs (A2B5−) both from pediatric and adult donors. Previous works by our group have shown that pediatric and adult human OL‐lineage cells differ on a transcriptomic level, with the indication that pediatric cells are more progenitor‐like (Luo et al. 2022). Our functional studies indicate a differential effect of fibrinogen in relation to OL lineage stage. Mature OLs (A2B5−), most apparent with adult donor cells, had an increased ensheathment capacity following fibrinogen exposure (Figure 2B). Further suggestions of this positive effect were found in our bulk RNAseq data, where GO‐BP terms showed enrichment for lipid metabolism pathways, as would be upregulated in OL‐mediated ensheathment and subsequent remyelination (O'Brien and Sampson 1965; Szuchet et al. 1983) (Figure 2G and File S3). These results indicate that both donor age and lineage stage underlie functional differences in the response of OLs to toxic molecules such as fibrinogen.

Previous work has shown that the effects of fibrinogen on rodent OL‐lineage cells are mediated by signaling via the BMP pathway, using a receptor (ACVR1) knockout model (Petersen et al. 2017). Our analysis of this pathway indicates differences between the primary and the iPSC‐derived OL‐lineage cells. Using immunocytochemistry, we observed a strong nuclear translocation of the downstream BMP effector molecule, pSMAD 1/5/9 in mature OLs; this response was less marked in the iPSC‐derived cells (Figure 3E). For both groups, we showed fibrinogen signaling inhibition using the ACVR1 inhibitor DMH1 (Figure 3A–D). Genes downstream of the BMP pathway were observed to be upregulated in the adult primary OLs (Figure 3F,G). On the other hand, the same analysis on early OL‐lineage cells showed non‐significant changes in the genes downstream of BMP signaling (Figure 3H,I). This, however, could be due to the observed upregulation of cell cycle pathways in response to fibrinogen (Figure 1 and Figure S5), where this effect may be more prominent in comparison to the downstream BMP genes. These observed responses can also be attributed to BMP receptor availability, as well as the presence of competing signaling molecules (Klumpe et al. 2022), as we could not deplete our cell media from all growth factors. Other pathways induced by fibrinogen would also need to be explored, to completely understand the mechanism of action in its entirety. We are aware that our low sample size is a limitation of our study, due to the nature of human primary cell culture work. Also, due to the variability of human genetic background, we have paired Bulk RNAseq samples for statistical analysis. We acknowledge that a greater sample size may circumvent this issue, as well as address biological sex differences. Future studies would be required to solidify the transcriptomic findings in the adult OLs, where sequencing in a time course could give more insight into the temporal effect of signaling and mechanism of functional response following exposure to fibrinogen.

Our study has shown that the effects of fibrinogen and BMP pathway activation are highly‐context dependent. In the perspective of development, studies have determined that BMP signaling has different effects dependent on developmental stage such as promoting neural progenitor cells to proliferate in early development (Mira et al. 2010) while promoting astrocytic and neuronal differentiation in later stages (Wang et al. 2014). Previous studies have also shown that BMP4 exposure of early OL lineage cells results in the expression of the astrocytic marker, GFAP, and an astrocyte‐like morphology (Grinspan et al. 2000). As seen in our iPSC‐OPCs, BMP4 signaling also has the capacity to retain cells in a progenitor‐like state (Zhang et al. 2013), seen via increased Ki67 expression (Figure S5), downregulation of OPC differentiation pathways (Figure 1G) and genes (Figure S4), and promotes a subset of cells to gain an astrocytic phenotype (Figure 1D,E and Figure S3). In other cell types, BMP signaling has been shown to promote cholesterol biosynthesis (Derwall et al. 2012). We have previously shown that various other toxic molecules have differential effects based on OL‐lineage stage (Mohammadnia et al. 2024; Fernandes et al. 2021), and studying the inherent differences between human cells across the OL‐lineage and the exact mechanisms dictating their differential response would require further exploration in our model. Furthermore, indirect activity of fibrinogen via other neural cell types (astrocytes, microglia), may add to the complexity of the role of fibrinogen in vivo and better reflect the in situ microenvironment, and would be of interest to explore in human primary cultures in a future study.

To confirm in situ relevance, we aimed to determine the co‐localization of fibrinogen with OL‐lineage cells in the MS brain. Using SOX10 as a pan‐lineage marker, we observed the co‐localization in various stages of MS lesion development as well as in NAWM (Figure 5). To our knowledge, this is the first exploration into fibrinogen co‐localization with OL‐lineage cells in the MS brain. The prevalence of BMPs (both ligands and receptors, as well as downstream signaling) has been previously reported in the MS brain (Deininger et al. 1995; Sabo et al. 2011; Costa et al. 2019). In situ studies have shown the correlation of BMPs with the level of inflammation MS lesions (Costa et al. 2019), and attribute this to both the presence of immune infiltrates as well as resident glial populations. Here we confirm these findings and show levels of downstream target genes in OL‐lineage cells differ based on lesion type (Figure 4). However, exactly how Fibrinogen and the BMP pathway is promoting remyelination in OLs and preventing differentiation in OPCs remains to be defined. Future studies addressing this would be important to confirm this present study as well as inform future drug development targets.

In the context of MS, one could speculate that the fibrinogen‐driven increased ensheathment by mature OLs could positively contribute to the remyelination process that occurs during early inflammatory demyelinating activity with ongoing breakdown of the BBB, and at the borders of chronic active plaques. Failure of subsequent remyelination would reflect a depletion of such cells, as evident in previously demyelinated regions of the plaque, that are not replenished by production of new OLs capable of remyelination due a block in oligodendroglial progenitor differentiation. This could reflect at least in part the effect of fibrinogen on OPCs. Whether other factors in the milieu of the MS lesion contribute to this differentiation block remains to be determined.

## Author Contributions

G.J.B., Q.‐L.C., C.W., J.P.A. and G.R.W.M. contributed substantially to the conception and design of the study. G.J.B., J.P.A., G.R.W.M. and A.M. drafted a significant portion of the manuscript or figures. G.J.B., Q.‐L.C., C.W., A.G., A.M.R.G., C.P., M.Y., J.S., V.E.C.P., J.A.H., R.W.R.D., M.S., S.E.J.Z., W.K., S.L., A.P., T.M.D., J.A.S. contributed to acquisition and analysis of data. A.T., L.C.M, and E.C. contributed to the development of potential immunohistochemical protocols for human tissue.

## Funding

This project was supported in part by a grant from MS Canada Grant Number 920522.

## Conflicts of Interest

The authors declare no conflicts of interest.

## Supporting information


**Data S1:** jnr70120‐sup‐0001‐FigureS1‐S8‐TableS1‐S4.docx.

**File S1:** Differentially Expressed Genes in iPSC‐OPCs. FACS‐sorted SOX10mO‐med cells were sent for BulkRNAseq. The read counts were run through DESeq2 for differential gene expression analysis between untreated and fibrinogen‐treated samples (*n* = 3). All significant genes based on the *p*‐value < 0.05 are in File S1. For the plots, additional filter of FC > 1.5 applied.

**File S2:** GO Term analysis results in iPSC‐OPCs.

**File S3:** Differentially Expressed genes in adult human primary OLs. Primary human OLs from two donors were treated with fibrinogen and sent for BulkRNAseq. Samples were analyzed in a pair‐wise manner as described in Mohammadnia et al. (2024). All significant genes based on the *p*‐value < 0.05 are in File S3.

**File S4:** GO‐BP, KEGG and Reactome term analysis on human primary OLs.

## Data Availability

The data that support the findings of this study are available from the corresponding author upon reasonable request.

## References

[jnr70120-bib-0001] Absinta, M. , D. Maric , M. Gharagozloo , et al. 2021. “A Lymphocyte‐Microglia‐Astrocyte Axis in Chronic Active Multiple Sclerosis.” Nature 597, no. 7878: 709–714. 34497421 10.1038/s41586-021-03892-7 PMC8719282

[jnr70120-bib-0002] Adams, R. A. , J. Bauer , M. J. Flick , et al. 2007. “The Fibrin‐Derived gamma377‐395 Peptide Inhibits Microglia Activation and Suppresses Relapsing Paralysis in Central Nervous System Autoimmune Disease.” Journal of Experimental Medicine 204, no. 3: 571–582. 17339406 10.1084/jem.20061931 PMC2137908

[jnr70120-bib-0003] Ahmad, U. , and J. L. Frederiksen . 2020. “Fibrinogen: A Potential Biomarker for Predicting Disease Severity in Multiple Sclerosis.” Multiple Sclerosis and Related Disorders 46: 102509. 32977072 10.1016/j.msard.2020.102509

[jnr70120-bib-0004] Alruwaili, M. , H. M. Al‐Kuraishy , A. Alexiou , et al. 2023. “Pathogenic Role of Fibrinogen in the Neuropathology of Multiple Sclerosis: A Tale of Sorrows and Fears.” Neurochemical Research 48, no. 11: 3255–3269. 37442896 10.1007/s11064-023-03981-1 PMC10514123

[jnr70120-bib-0005] Barbar, L. , T. Jain , M. Zimmer , et al. 2020. “CD49f Is a Novel Marker of Functional and Reactive Human iPSC‐Derived Astrocytes.” Neuron 107, no. 3: 436–453. 32485136 10.1016/j.neuron.2020.05.014 PMC8274549

[jnr70120-bib-0006] Bardehle, S. , V. A. Rafalski , and K. Akassoglou . 2015. “Breaking Boundaries‐Coagulation and Fibrinolysis at the Neurovascular Interface.” Frontiers in Cellular Neuroscience 9: 354. 26441525 10.3389/fncel.2015.00354 PMC4584986

[jnr70120-bib-0007] Broux, B. , S. Zandee , E. Gowing , et al. 2020. “Interleukin‐26, Preferentially Produced by T(H)17 Lymphocytes, Regulates CNS Barrier Function.” Neurology Neuroimmunology & Neuroinflammation 7, no. 6: e870. 10.1212/NXI.0000000000000870 PMC7428369 32788322

[jnr70120-bib-0008] Browaeys, R. , W. Saelens , and Y. Saeys . 2020. “NicheNet: Modeling Intercellular Communication by Linking Ligands to Target Genes.” Nature Methods 17, no. 2: 159–162. 31819264 10.1038/s41592-019-0667-5

[jnr70120-bib-0009] Budzynski, A. Z. 1986. “Fibrinogen and Fibrin: Biochemistry and Pathophysiology.” Critical Reviews in Oncology/Hematology 6, no. 2: 97–146. 2878736 10.1016/s1040-8428(86)80019-1

[jnr70120-bib-0010] Çiçekli, E. , S. Sayan , and D. Kotan . 2022. “Availability of Fibrinogen/Albumin Ratio in MS Attack.” Multiple Sclerosis and Related Disorders 60: 103674. 35290899 10.1016/j.msard.2022.103674

[jnr70120-bib-0011] Costa, C. , H. Eixarch , E. Martínez‐Sáez , et al. 2019. “Expression of Bone Morphogenetic Proteins in Multiple Sclerosis Lesions.” American Journal of Pathology 189, no. 3: 665–676. 30553833 10.1016/j.ajpath.2018.11.007

[jnr70120-bib-0012] Davalos, D. , K. R. Mahajan , and B. D. Trapp . 2019. “Brain Fibrinogen Deposition Plays a Key Role in MS Pathophysiology ‐ Yes.” Multiple Sclerosis 25, no. 11: 1434–1435. 31315512 10.1177/1352458519852723 PMC6750992

[jnr70120-bib-0013] Deininger, M. , R. Meyermann , and H. Schluesener . 1995. “Detection of Two Transforming Growth Factor‐Beta‐Related Morphogens, Bone Morphogenetic Proteins‐4 and ‐5, in RNA of Multiple Sclerosis and Creutzfeldt‐Jakob Disease Lesions.” Acta Neuropathologica 90, no. 1: 76–79. 7572083 10.1007/BF00294462

[jnr70120-bib-0014] Derwall, M. , R. Malhotra , C. S. Lai , et al. 2012. “Inhibition of Bone Morphogenetic Protein Signaling Reduces Vascular Calcification and Atherosclerosis.” Arteriosclerosis, Thrombosis, and Vascular Biology 32, no. 3: 613–622. 22223731 10.1161/ATVBAHA.111.242594 PMC3679546

[jnr70120-bib-0015] Dhaeze, T. , L. Tremblay , C. Lachance , et al. 2019. “CD70 Defines a Subset of Proinflammatory and CNS‐Pathogenic T(H)1/T(H)17 Lymphocytes and Is Overexpressed in Multiple Sclerosis.” Cellular & Molecular Immunology 16, no. 7: 652–665. 30635649 10.1038/s41423-018-0198-5 PMC6804668

[jnr70120-bib-0016] Esmonde‐White, C. , M. Yaqubi , P. A. Bilodeau , et al. 2019. “Distinct Function‐Related Molecular Profile of Adult Human A2B5‐Positive Pre‐Oligodendrocytes Versus Mature Oligodendrocytes.” Journal of Neuropathology and Experimental Neurology 78, no. 6: 468–479. 31058285 10.1093/jnen/nlz026

[jnr70120-bib-0017] Fernandes, M. G. F. , J. X. X. Luo , Q.‐L. Cui , et al. 2021. “Age‐Related Injury Responses of Human Oligodendrocytes to Metabolic Insults: Link to BCL‐2 and Autophagy Pathways.” Communications Biology 4, no. 1: 20. 33398046 10.1038/s42003-020-01557-1 PMC7782481

[jnr70120-bib-0018] Grinspan, J. B. , E. Edell , D. F. Carpio , et al. 2000. “Stage‐Specific Effects of Bone Morphogenetic Proteins on the Oligodendrocyte Lineage.” Journal of Neurobiology 43, no. 1: 1–17. 10756062

[jnr70120-bib-0019] Heldin, C. H. , K. Miyazono , and P. ten Dijke . 1997. “TGF‐Beta Signalling From Cell Membrane to Nucleus Through SMAD Proteins.” Nature 390, no. 6659: 465–471. 9393997 10.1038/37284

[jnr70120-bib-0020] Jagielska, A. , A. L. Norman , G. Whyte , K. J. Vliet , J. Guck , and R. J. Franklin . 2012. “Mechanical Environment Modulates Biological Properties of Oligodendrocyte Progenitor Cells.” Stem Cells and Development 21, no. 16: 2905–2914. 22646081 10.1089/scd.2012.0189 PMC5915215

[jnr70120-bib-0021] Klumpe, H. E. , M. A. Langley , J. M. Linton , C. J. Su , Y. E. Antebi , and M. B. Elowitz . 2022. “The Context‐Dependent, Combinatorial Logic of BMP Signaling.” Cell Systems 13, no. 5: 388–407. 35421361 10.1016/j.cels.2022.03.002 PMC9127470

[jnr70120-bib-0022] Kuhlbrodt, K. , B. Herbarth , E. Sock , I. Hermans‐Borgmeyer , and M. Wegner . 1998. “Sox10, a Novel Transcriptional Modulator in Glial Cells.” Journal of Neuroscience 18, no. 1: 237–250. 9412504 10.1523/JNEUROSCI.18-01-00237.1998 PMC6793382

[jnr70120-bib-0023] Lee, N. J. , S. K. Ha , P. Sati , et al. 2018. “Spatiotemporal Distribution of Fibrinogen in Marmoset and Human Inflammatory Demyelination.” Brain 141, no. 6: 1637–1649. 29688408 10.1093/brain/awy082 PMC5972667

[jnr70120-bib-0024] Leong, S. Y. , V. T. Rao , J. M. Bin , et al. 2014. “Heterogeneity of Oligodendrocyte Progenitor Cells in Adult Human Brain.” Annals of Clinical and Translational Neurology 1, no. 4: 272–283. 25590039 10.1002/acn3.55 PMC4292744

[jnr70120-bib-0025] Love, M. I. , W. Huber , and S. Anders . 2014. “Moderated Estimation of Fold Change and Dispersion for RNA‐Seq Data With DESeq2.” Genome Biology 15, no. 12: 550. 25516281 10.1186/s13059-014-0550-8 PMC4302049

[jnr70120-bib-0027] Luo, J. X. X. , Q. L. Cui , M. Yaqubi , et al. 2022. “Human Oligodendrocyte Myelination Potential; Relation to Age and Differentiation.” Annals of Neurology 91, no. 2: 178–191. 34952986 10.1002/ana.26288

[jnr70120-bib-0028] Mabie, P. C. , M. F. Mehler , R. Marmur , A. Papavasiliou , Q. Song , and J. A. Kessler . 1997. “Bone Morphogenetic Proteins Induce Astroglial Differentiation of Oligodendroglial‐Astroglial Progenitor Cells.” Journal of Neuroscience 17, no. 11: 4112–4120. 9151728 10.1523/JNEUROSCI.17-11-04112.1997 PMC6573548

[jnr70120-bib-0029] Marik, C. , P. A. Felts , J. Bauer , H. Lassmann , and K. J. Smith . 2007. “Lesion Genesis in a Subset of Patients With Multiple Sclerosis: A Role for Innate Immunity?” Brain 130, no. 11: 2800–2815. 17956913 10.1093/brain/awm236 PMC2981817

[jnr70120-bib-0030] McQuaid, S. , P. Cunnea , J. McMahon , and U. Fitzgerald . 2009. “The Effects of Blood‐Brain Barrier Disruption on Glial Cell Function in Multiple Sclerosis.” Biochemical Society Transactions 37, no. Pt 1: 329–331. 19143657 10.1042/BST0370329

[jnr70120-bib-0031] Mira, H. , Z. Andreu , H. Suh , et al. 2010. “Signaling Through BMPR‐IA Regulates Quiescence and Long‐Term Activity of Neural Stem Cells in the Adult Hippocampus.” Cell Stem Cell 7, no. 1: 78–89. 20621052 10.1016/j.stem.2010.04.016

[jnr70120-bib-0032] Mohammadnia, A. , Q.‐L. Cui , C. Weng , et al. 2024. “Age‐Dependent Effects of Metformin on Human Oligodendrocyte Lineage Cell Ensheathment Capacity.” Brain Communications 6, no. 2: fcae109. 38601917 10.1093/braincomms/fcae109 PMC11005772

[jnr70120-bib-0033] Mohammadnia, A. , D. Dansu , Q.‐L. Cui , et al. 2025. “Epigenomic Regulation of Human Oligodendrocyte Myelination Properties—Relation to Age and Lineage.” bioRxi 9, no. 4: 674347.

[jnr70120-bib-0055] Moore, G. R. W. , and C. Stadelmann‐Nessler . 2015. “Demyelinating Diseases.” In Greenfield's Neuropathology, edited by Love S. , H. Budka , J. W. Ironside , and A. Perry , 9th ed., 1333–1334. CRC Press.

[jnr70120-bib-0034] Mosesson, M. W. 2005. “Fibrinogen and Fibrin Structure and Functions.” Journal of Thrombosis and Haemostasis 3, no. 8: 1894–1904. 16102057 10.1111/j.1538-7836.2005.01365.x

[jnr70120-bib-0035] O'Brien, J. S. , and E. L. Sampson . 1965. “Lipid Composition of the Normal Human Brain: Gray Matter, White Matter, and Myelin.” Journal of Lipid Research 6, no. 4: 537–544. 5865382

[jnr70120-bib-0036] Pernin, F. , Q.‐L. Cui , A. Mohammadnia , et al. 2024. “Regulation of Stress Granule Formation in Human Oligodendrocytes.” Nature Communications 15, no. 1: 1524. 10.1038/s41467-024-45746-6 PMC10876533 38374028

[jnr70120-bib-0037] Petersen, M. A. , J. K. Ryu , and K. Akassoglou . 2018. “Fibrinogen in Neurological Diseases: Mechanisms, Imaging and Therapeutics.” Nature Reviews. Neuroscience 19, no. 5: 283–301. 29618808 10.1038/nrn.2018.13 PMC6743980

[jnr70120-bib-0038] Petersen, M. A. , J. K. Ryu , K. J. Chang , et al. 2017. “Fibrinogen Activates BMP Signaling in Oligodendrocyte Progenitor Cells and Inhibits Remyelination After Vascular Damage.” Neuron 96, no. 5: 1003–1012. 29103804 10.1016/j.neuron.2017.10.008 PMC5851281

[jnr70120-bib-0039] Piscopo, V. E. C. , A. Chapleau , G. J. Blaszczyk , et al. 2024. “The Use of a SOX10 Reporter Toward Ameliorating Oligodendrocyte Lineage Differentiation From Human Induced Pluripotent Stem Cells.” Glia 72, no. 6: 1165–1182. 38497409 10.1002/glia.24524

[jnr70120-bib-0040] Polman, C. H. , S. C. Reingold , B. Banwell , et al. 2011. “Diagnostic Criteria for Multiple Sclerosis: 2010 Revisions to the McDonald Criteria.” Annals of Neurology 69, no. 2: 292–302. 21387374 10.1002/ana.22366 PMC3084507

[jnr70120-bib-0041] Reich, M. , T. Liefeld , J. Gould , J. Lerner , P. Tamayo , and J. P. Mesirov . 2006. “GenePattern 2.0.” Nature Genetics 38, no. 5: 500–501. 16642009 10.1038/ng0506-500

[jnr70120-bib-0042] Ruffini, F. , N. Arbour , M. Blain , A. Olivier , and J. P. Antel . 2004. “Distinctive Properties of Human Adult Brain‐Derived Myelin Progenitor Cells.” American Journal of Pathology 165, no. 6: 2167–2175. 15579458 10.1016/S0002-9440(10)63266-X PMC1618716

[jnr70120-bib-0043] Ryu, J. K. , M. A. Petersen , S. G. Murray , et al. 2015. “Blood Coagulation Protein Fibrinogen Promotes Autoimmunity and Demyelination via Chemokine Release and Antigen Presentation.” Nature Communications 6: 8164. 10.1038/ncomms9164 PMC4579523 26353940

[jnr70120-bib-0044] Sabo, J. K. , T. D. Aumann , D. Merlo , T. J. Kilpatrick , and H. S. Cate . 2011. “Remyelination Is Altered by Bone Morphogenic Protein Signaling in Demyelinated Lesions.” Journal of Neuroscience 31, no. 12: 4504–4510. 21430151 10.1523/JNEUROSCI.5859-10.2011 PMC6622914

[jnr70120-bib-0045] Schachtrup, C. , J. K. Ryu , M. J. Helmrick , et al. 2010. “Fibrinogen Triggers Astrocyte Scar Formation by Promoting the Availability of Active TGF‐Beta After Vascular Damage.” Journal of Neuroscience 30, no. 17: 5843–5854. 20427645 10.1523/JNEUROSCI.0137-10.2010 PMC2871011

[jnr70120-bib-0046] Szuchet, S. , S. H. Yim , and S. Monsma . 1983. “Lipid Metabolism of Isolated Oligodendrocytes Maintained in Long‐Term Culture Mimics Events Associated With Myelinogenesis.” Proceedings of the National Academy of Sciences of the United States of America 80, no. 22: 7019–7023. 6580624 10.1073/pnas.80.22.7019 PMC390118

[jnr70120-bib-0047] Vos, C. M. , J. J. Geurts , L. Montagne , et al. 2005. “Blood‐Brain Barrier Alterations in Both Focal and Diffuse Abnormalities on Postmortem MRI in Multiple Sclerosis.” Neurobiology of Disease 20, no. 3: 953–960. 16039866 10.1016/j.nbd.2005.06.012

[jnr70120-bib-0048] Wang, R. N. , J. Green , Z. Wang , et al. 2014. “Bone Morphogenetic Protein (BMP) Signaling in Development and Human Diseases.” Genes and Diseases 1, no. 1: 87–105. 25401122 10.1016/j.gendis.2014.07.005 PMC4232216

[jnr70120-bib-0049] Wen, T. , and Z. Zhang . 2023. “Cellular Mechanisms of Fibrin (ogen): Insight From Neurodegenerative Diseases.” Frontiers in Neuroscience 17: 1197094. 37529232 10.3389/fnins.2023.1197094 PMC10390316

[jnr70120-bib-0050] Yates, R. L. , M. M. Esiri , J. Palace , B. Jacobs , R. Perera , and G. C. DeLuca . 2017. “Fibrin (ogen) and Neurodegeneration in the Progressive Multiple Sclerosis Cortex.” Annals of Neurology 82, no. 2: 259–270. 28719020 10.1002/ana.24997

[jnr70120-bib-0051] Yeung, M. S. , S. Zdunek , O. Bergmann , et al. 2014. “Dynamics of Oligodendrocyte Generation and Myelination in the Human Brain.” Cell 159, no. 4: 766–774. 25417154 10.1016/j.cell.2014.10.011

[jnr70120-bib-0052] Zhang, J. , T. Fei , Z. Li , G. Zhu , L. Wang , and Y. G. Chen . 2013. “BMP Induces Cochlin Expression to Facilitate Self‐Renewal and Suppress Neural Differentiation of Mouse Embryonic Stem Cells.” Journal of Biological Chemistry 288, no. 12: 8053–8060. 23344953 10.1074/jbc.M112.433995 PMC3605624

[jnr70120-bib-0053] Zierfuss, B. , C. Larochelle , and A. Prat . 2024. “Blood‐Brain Barrier Dysfunction in Multiple Sclerosis: Causes, Consequences, and Potential Effects of Therapies.” Lancet Neurology 23, no. 1: 95–109. 38101906 10.1016/S1474-4422(23)00377-0

[jnr70120-bib-0054] Ziliotto, N. , F. Bernardi , D. Jakimovski , and R. Zivadinov . 2019. “Coagulation Pathways in Neurological Diseases: Multiple Sclerosis.” Frontiers in Neurology 10: 409. 31068896 10.3389/fneur.2019.00409 PMC6491577

