# Supplementary material for: Age and Maturation Stage Linked Consequences of Fibrinogen on Human Oligodendroglia
Source: J Neurosci Res. 2026 Mar 22;104(3):e70120. doi: 10.1002/jnr.70120 (PMC13006721; doi:10.1002/jnr.70120)
Supplement: Supplementary file 1 — Data S1: jnr70120‐sup‐0001‐FigureS1‐S8‐TableS1‐S4.docx. [file JNR-104-e70120-s003.docx]

**
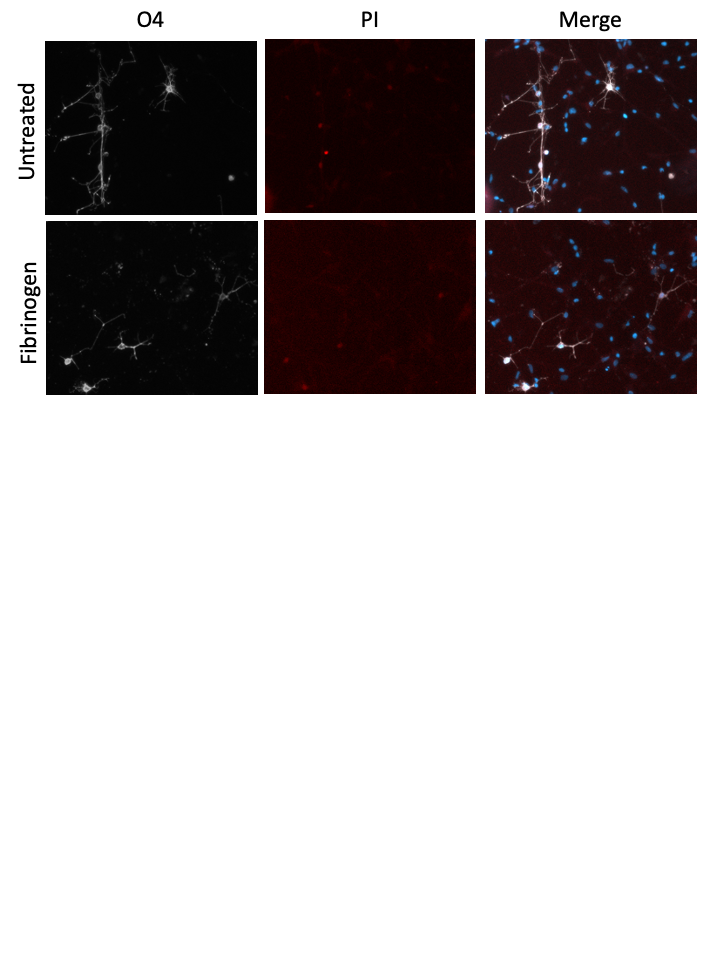
**

**Figure S1. Representative images of hiPSC-OPC cultures.** Following 21-day differentiation and 4-Day treatment with fibrinogen. Propidium iodide in red, stains for dead cells, O4 in white stains for OL-lineage cells, and Hoechst stains for nuclei in blue.

**
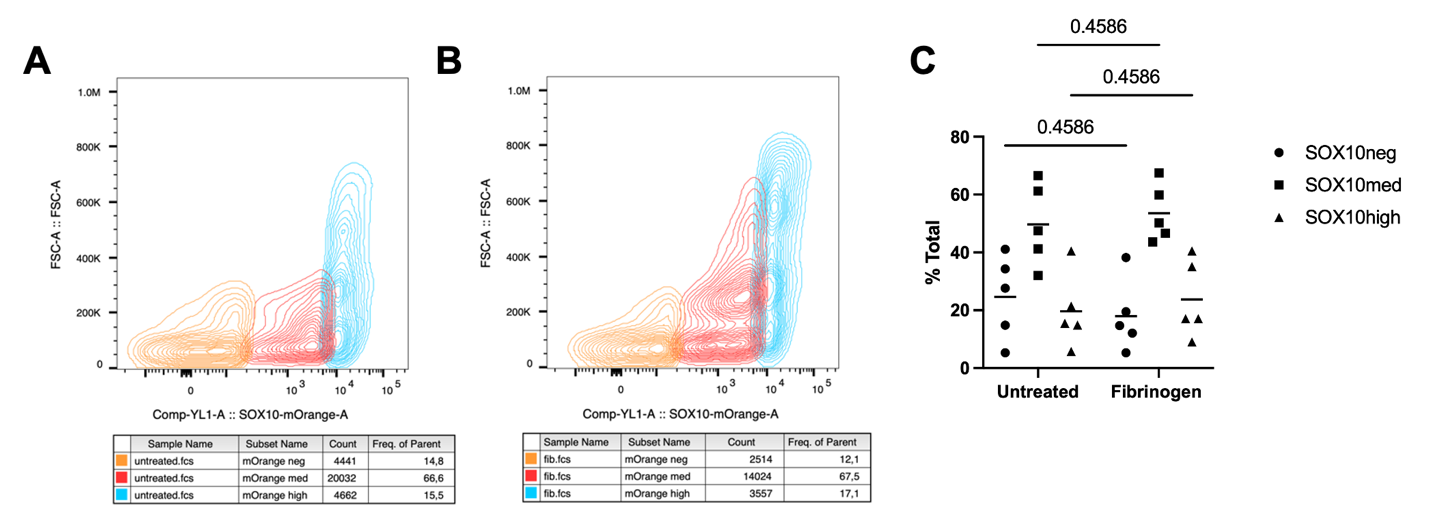
**

**Figure S2. Proportion of SOX10mO subgroups changes with Fibrinogen treatment. A&B.** Representative contour plots of mOrange subgroups (A) untreated and (B) fibrinogen-treated samples acquired by flow cytometry. Reporter fluorescence intensity has previously been shown to correlate with OL-lineage stage (Piscopo et al., 2024). **C.** Proportion of live cells in each reporter category based on fluorescence intensity (negative, medium, high). Increasing trend in the proportion of SOX10-med cells observed, FDR corrected p-values insignificant.

**
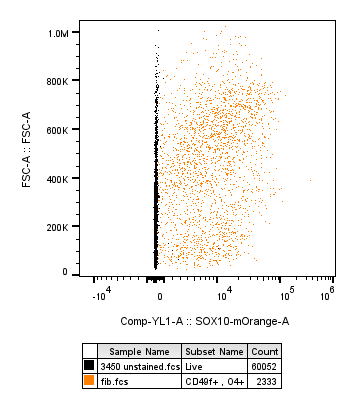
Figure S3. mOrange signal in hybrid OPCs.** Following 4-day treatment of fibrinogen, data acquired by flow cytometry. CD49f+O4+ cells express mOrange (orange) at a higher fluorescence intensity in comparison to non-reporter cells (black).

**
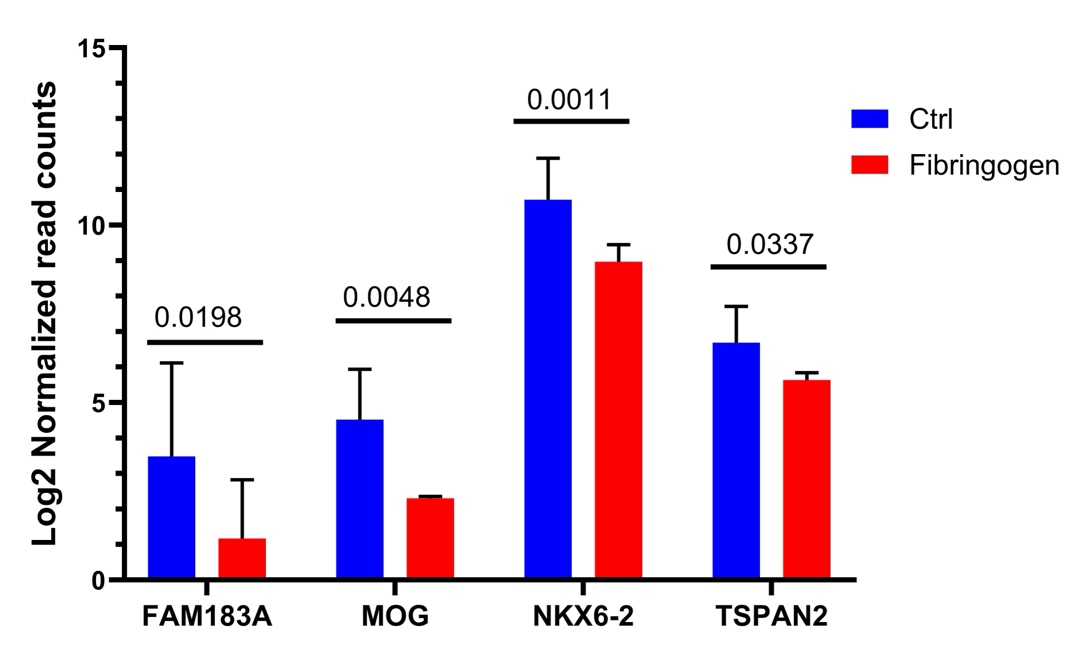
**

Ctrl

Fibrinogen

**Figure S4. OPC-differentiation related genes which are significantly downregulated with Fibrinogen treatment of hiPSC-OPCs.**  Log2 normalized read counts, output of DEseq2. Resulting p-values plotted following ttest.

**
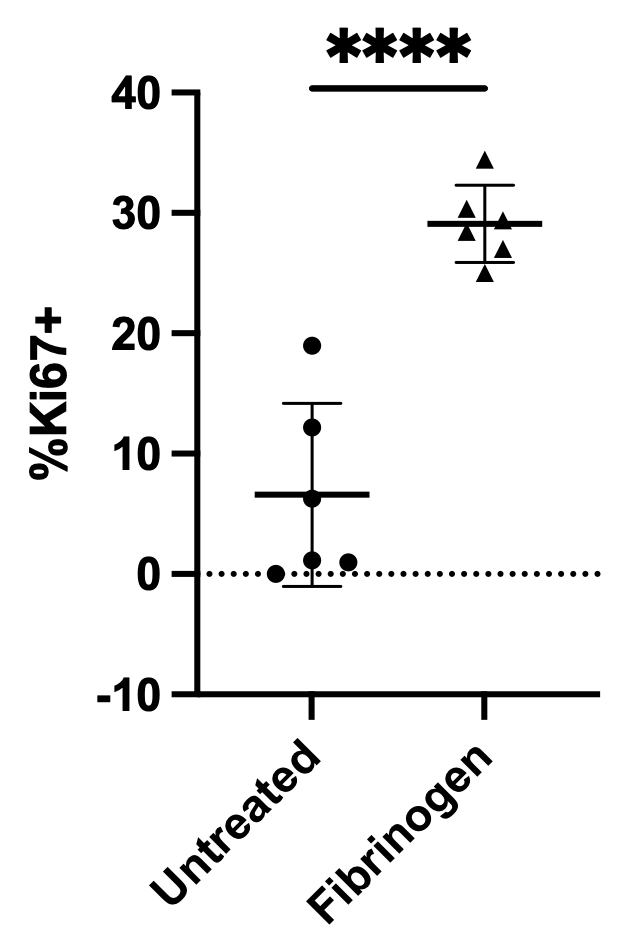
Figure S5. Proliferation in fibrinogen treated OPC cultures.** Proportion of Ki67+ nuclei quantified in iPSC-OPC cultures treated with fibrinogen. p < 0.0001. n=6 replicates, bars indicate SD.


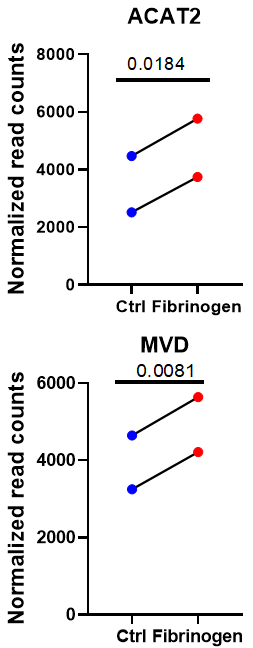

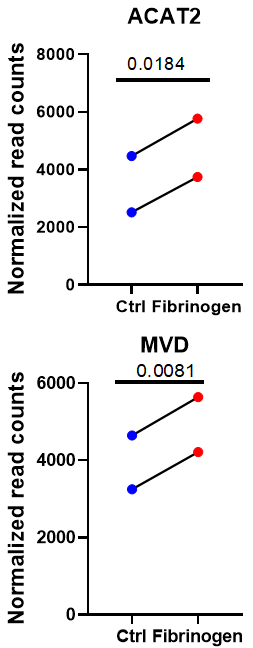


**A**

**B**

**Figure S6. Genes related to lipid synthesis which are significantly upregulated with Fibrinogen treatment, in Adult A2B5- Human primary OLs. A-B**. Examples of upregulated genes related to lipid synthesis. Statistics performed with a paired t-test, with treated and untreated cells in the same replicate from the same human donor. In all plots, blue represents control samples, and red represents fibrinogen-treated samples.

**Figure S7. BMP-related genes upregulated in OLs treated with Fibrinogen.**


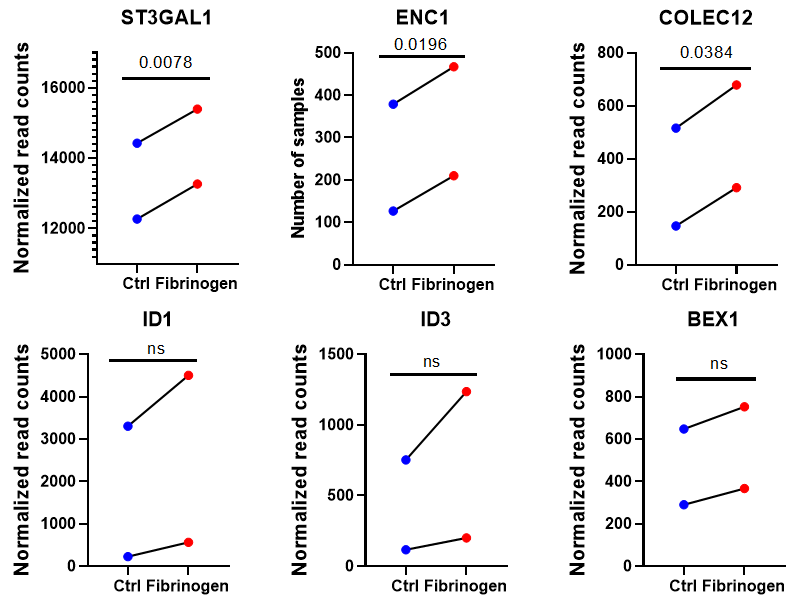

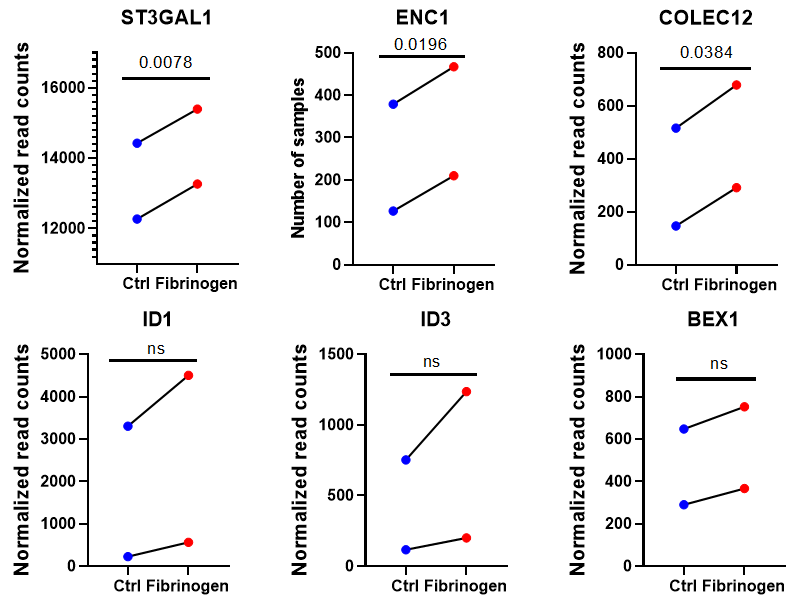


**A**

**B**

**C**

**D**

**E**

**F**

**A-F.** Examples of BMP4 target genes in human primary OLs showing upregulation upon treatment with fibrinogen (2.5 mg/mL, 2 days). Paired t-tests were applied to assess significance levels in all plots for human primary OLs. In all plots, blue represents control samples, and red represents fibrinogen-treated samples.

**
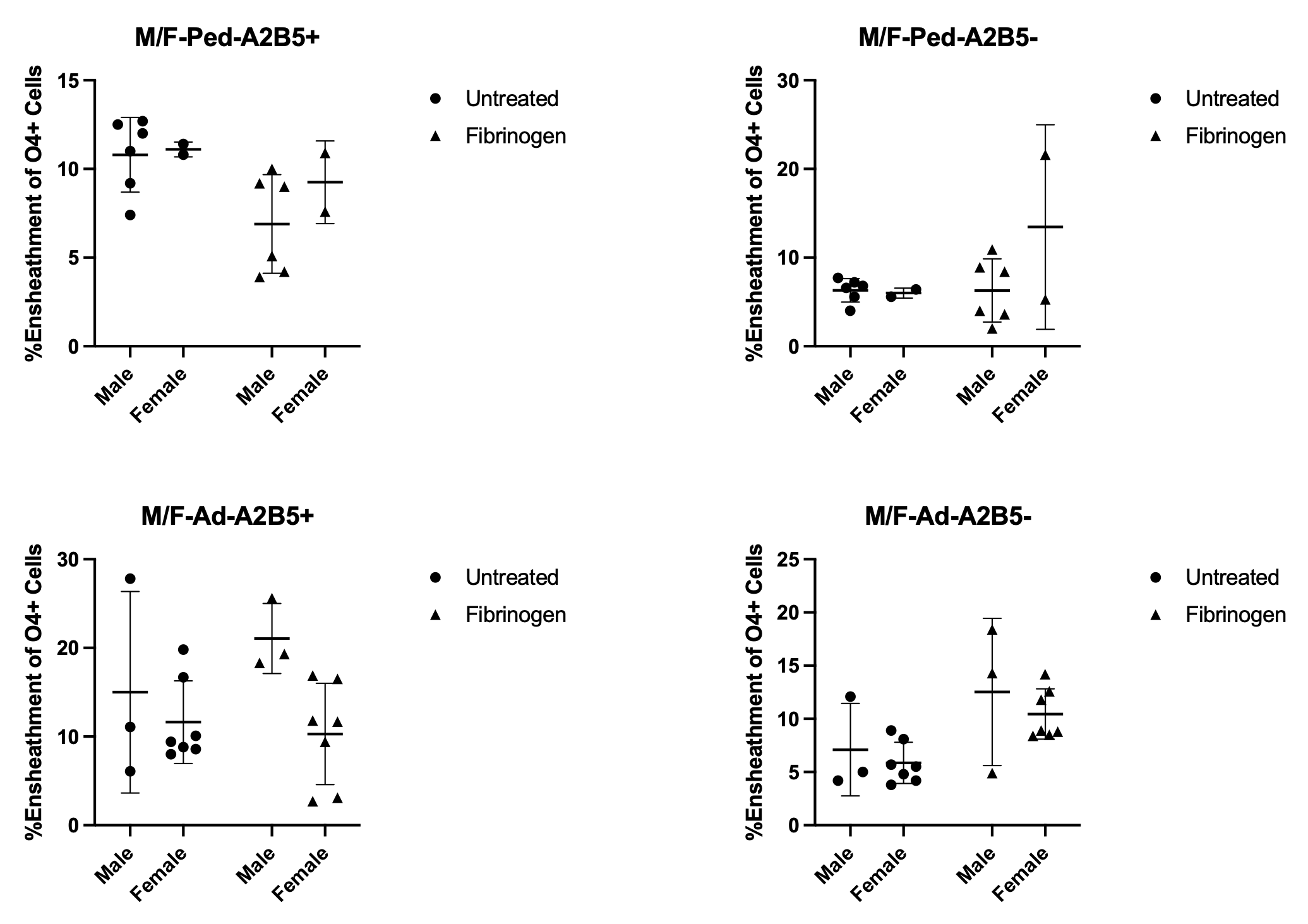
Figure S8. Disaggregated data of human primary OL ensheathment assay, based on biological sex of donor.** Datapoints represented in main figures 2B & 2C. Bars represent SD.

**Table S1. Primary OL Donor information.**

| **Age Group** | **Number of Samples** | **Average Age (years)** | **Median Age (years)** | **Min Age (years)** | **Max Age (years)** | **# Female (%)** |
| --- | --- | --- | --- | --- | --- | --- |
| Pediatric | 8 | 11 | 12 | 2 | 18 | 2 (25%) |
| Adult | 10 | 50 | 50 | 26 | 65 | 7 (70%) |

**Table S2. iPSC Line Information**

| **Cell Line Name** | **Sex** | **Patient Age (years)** | **Material Source** | **Type of Reprogramming** |
| --- | --- | --- | --- | --- |
| AIW002-02 | M | 37 | PBMC | Retrovirus |
| 3450 | M | 37 | PBMC | Episomal |
| SOX10mOrange (3450) | M | 37 | PBMC | Episomal |

Abbreviations: M=Male, PBMC= Peripheral Blood Mononuclear Cells.

**Table S3. Tissue Donor information.**

| **Section** | **Age**  **(years)** | **Sex** | **Disease Type** | **EDSS** | **Disease Duration** | **Post-mortem**  **interval** |
| --- | --- | --- | --- | --- | --- | --- |
| MS Plaque 1 | 48 | M | SPMS | 8.5 | 6 years | N/A |
| MS Plaque 2 | 60 | F | SPMS | 8.5 | 28 years | 2.5 hrs |
| MS Plaque 3 | 44 | F | RRMS | 7 | 13 years | 5 hr |

Abbreviations: EDSS=Expanded Disability Status Scale, F= Female, M=Male, MS=Multiple Sclerosis, N/A=Not Available, RRMS=Relapsing-remitting MS, SPMS=Secondary Progressive MS.

**Table S4. Antibodies and Reagents**

| **Antibody** | **Supplier** | **Identifier** |
| --- | --- | --- |
| goat anti-SOX10 | R&D systems, Oakville ON | AF2864 |
| rabbit anti-Fibrinogen | Agilent, Santa Clara CA | A0080 |
| isotype control polyclonal rabbit | BioLegend, San Diego CA | 910801 |
| isotype control goat IgG | R&D systems, Oakville ON | AB 108-C |
| O4-APC | Miltenyi, Auburn CA | 130-119-982 |
| Human TruStain FcX Fc Receptor Blocking Solution | BioLegend, San Diego CA | 422302 |
| LIVE/DEAD Fixable Aqua Dead Cell Stain Kit, for 405nm excitation | Invitrogen, Waltham MA | L34957 |
| CD49f-PE-Dazzle | BioLegend, San Diego CA | 313626 |
| mouse IgM anti-O4 | R&D, Oakville ON | MAB1326 |
| rabbit anti-pSMAD1/5/9 | Cell Signalling Technology, Danvers MA | 13820S |
| rat anti-Ki67 | Invitrogen, Waltham MA | 14-5698-82 |
